# Siderophores as tools and treatments

**DOI:** 10.1038/s44259-024-00053-4

**Published:** 2024-12-05

**Authors:** Á. Tamás Gräff, Sarah M. Barry

**Affiliations:** Department of Chemistry, Faculty of Natural, Mathematical and Engineering Sciences, King’s College London, Britannia House, London, SE1 1DB UK

**Keywords:** Chemical tools, Natural product synthesis, Transporters

## Abstract

In the search for iron, an essential element in many biochemical processes, microorganisms biosynthesise dedicated chelators, known as siderophores, to sequester iron from their environment and actively transport the siderophore complex into the cell. This process has been implicated in bacterial pathogenesis and exploited through siderophore-antibiotic conjugates as a method for selective antibiotic delivery. Here we review this Trojan-horse approach including design considerations and potential in diagnostics and infection imaging.

## Introduction

Antimicrobial resistance (AMR) is a growing global health crisis. The growth in Gram-negative antibiotic-resistant infections and extremely multidrug resistant (XMDR) infections is particularly problematic^1^. Tackling this problem requires a multifaceted approach involving improved management of existing antibiotics and development of new therapeutics^2^. However, we still have significant gaps in our knowledge on fundamental microbial physiology and how, for example, nutrient flux, secondary metabolism, etc. intersect with pathogenesis. Of particular interest is the homoeostasis of essential, but potentially toxic, micronutrients including metal ions.

Ferric ions (Fe^3+^) are vital to many biochemical processes (gas transport, electron transport, etc.) as they are core components of enzyme cofactors e.g. iron–sulfur clusters, haem. However, free iron generates reactive hydroxyl radicals through the Fenton process, thus the labile iron pool in the cell must be carefully managed^3,4^. Furthermore, iron uptake and storage are significant biochemical challenges for many organisms because at neutral pH, free iron forms highly insoluble ferric oxide hydrates, limiting bioavailable iron to roughly 10^−18^ M^5,6^. In a mammalian host-pathogen environment, iron availability is particularly acute, as the circulatory system has a free ferric ion concentration around 10^−^^24^ M, which is below the threshold required for most microorganisms to grow^7^.

However, the iron acquisition challenge in microorganisms is in part overcome by their ability to biosynthesise complex, specialised metabolites to sequester iron from their hosts^8^. Once biosynthesised, these bespoke iron-chelating small molecules, known as siderophores, are secreted into the host environment to sequester iron^9^. The ferric complex is subsequently actively transported into the cell. The success of siderophores relies both on their incredible affinity for iron and the selective recognition and active transport of the resulting iron complexes across the bacterial cell membrane^9^. Several siderophores have been identified as potential virulence factors in pathogenicity (e.g. salmochelin, pyochelin) and as siderophores are not produced in mammals, proteins involved in their production have been seen as antibiotic targets^10^. Thus, the study of siderophore biosynthesis, pathway regulation and siderophore uptake have become significant research topics.

### Siderophores: biological role, biosynthesis and uptake

**Fig. 1:**
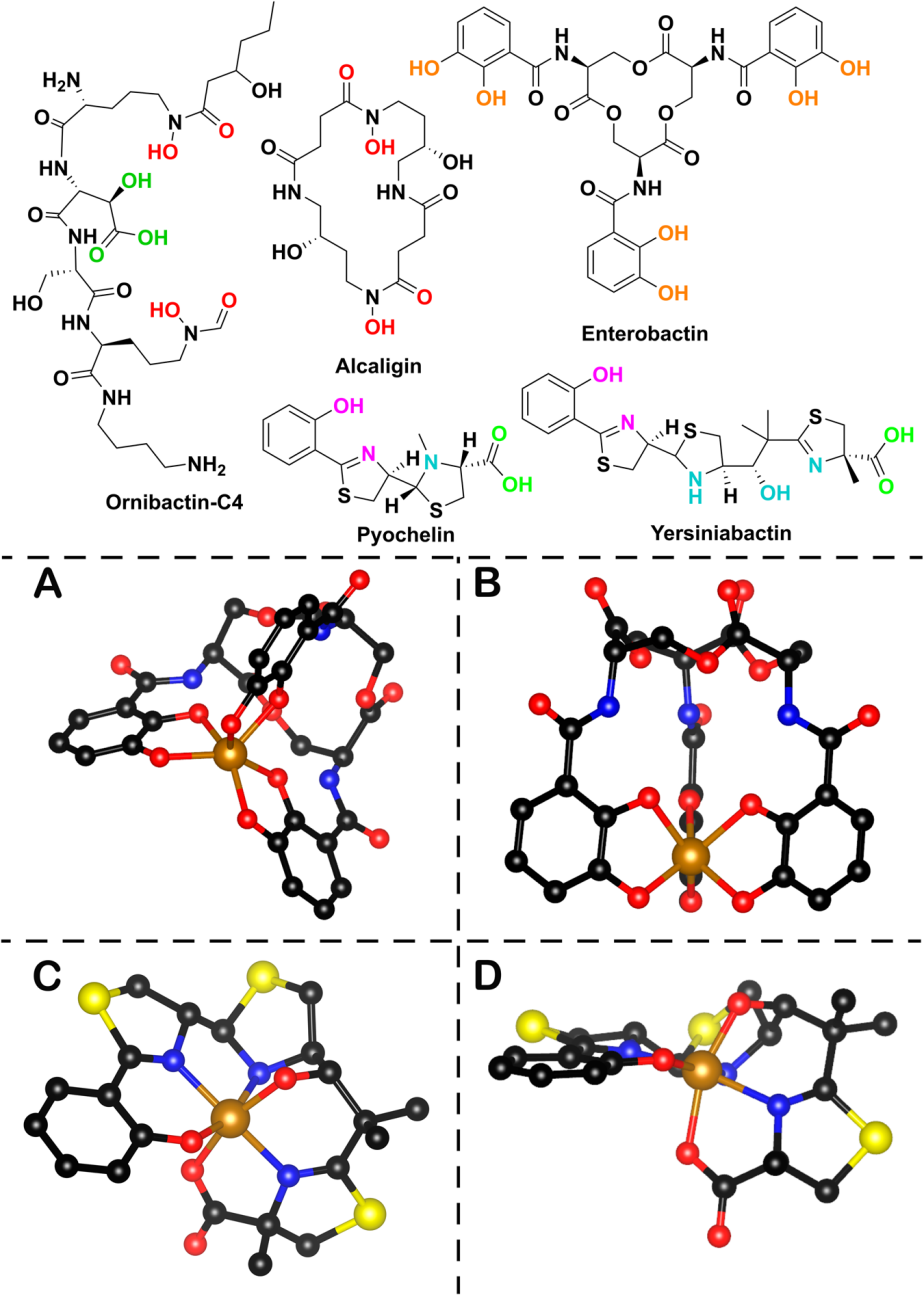
Siderophores and their chelation modes. Top: examples of natural product bacterial siderophores alcaligin (*Alcaligenes denitrificans*, *Bordetella* sp); enterobactin (*E. coli*), pyochelin (*Pseudomonas aeruginosa*), yersiniabactin (*Yersinia* sp), ornibactin (*Burkholderia* sp). The different colours correspond to different chelating moieties. Bottom: **A** Side view of metal (Fe^III^) chelated enterobactin (PDB 6Q5E). **B** Bottom view of enterobactin-Fe^III^ complex. **C** Front view of metal (Fe^III^) chelated yersiniabactin (CCDC 619878). **D** Side view of yersiniabactin-Fe^III^ complex (the images were made using Chemdraw Professional version 22.2.0.3300 and VESTA 3)^155^.

Microbial siderophores consist of several classes based on both their chelating moieties and their biosynthetic origin. The most common chelating moieties are catechols and phenolates, as exemplified by enterobactin and salmochelin produced by *Escherichia coli* (*E. coli*) and *Salmonella* sp respectively, and hydroxamic acids and carboxylates as shown in alcaligin and ornibactin (Fig. 1). There are also many siderophores that display mixed modes of chelation such as yersiniabactin. Siderophore iron affinity is often described using the logarithm of the formation constant (logK_f_) which for representative ferric complexes ranges between 25.3 and 49.0^9^. However this may not be necessarily the best way to compare the different chelators, due to the difference between the pH sensitivity of the chelating groups, the different denticity of the ligands and other steric factors^11^. pFe^III^, analogous to pH, provides a more general measurement, as it is based on the negative logarithm of the non-chelated ferric hexahydrate ions in specific experimental conditions (usually pH = 7.4, total [Fe] = 1 µm, total [L] = 10 µm). Siderophores have pFe^III^ measurements in the range of 20.0–35.5^9,12^.

Siderophore biosynthetic pathways are typically encoded by biosynthetic gene clusters and while some are expressed constitutively, most are regulated by iron and expressed only in low iron concentrations^6^. Typically, siderophore discovery and isolation is thus facilitated by growing strains in iron-deficient minimal media. The biosynthetic origin of siderophores varies, with enterobactin, salmochelin and mycobactin being non-ribosomal peptides (NRPS), and many of the citrate-based siderophores such as aerobactin, petrobactin and alcaligin resulting from so-called NRPS independent synthetase (NIS) pathways^13–15^. In all cases, bespoke, non-proteinogenic amino acids are biosynthesised in a pathway-specific manner to create the chelating moieties within these structures.

Following biosynthesis, siderophores are secreted into the environment. On chelation to iron, the resulting ferric complex is recognised by a receptor on the cell surface. These receptor/transporter systems are generally encoded in the same gene clusters as the biosynthetic genes and are usually siderophore specific. However, it is known that bacteria are capable of sharing or competing for the siderophores of other bacterial strains, creating complex uptake and exchange networks in microbial communities^16^.

The siderophore uptake machinery differs between Gram-negative and Gram-positive bacteria. Ferric-siderophore complex recognition and transport is somewhat poorly understood and few examples of siderophore receptor/transporters have been structurally characterised^17–23^.

**Fig. 2:**
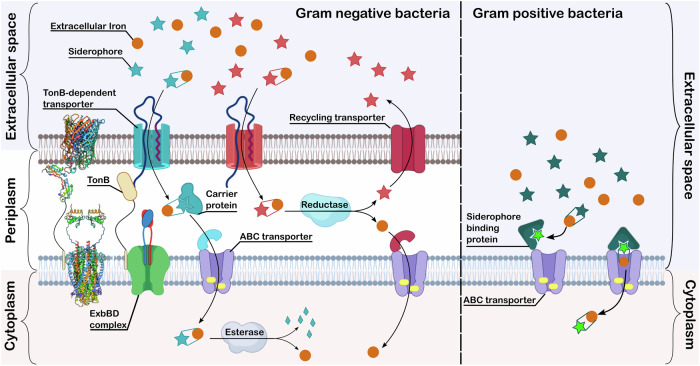
Siderophore uptake systems in Gram-negative and Gram-positive bacteria. The protein structures on the left-hand side of the figure are representative structures of a TonB-dependent transporter (PDB 1FEP)^17^, a *C*-terminal part of TonB (PDB 1U07)^18^ and an ExdBD complex (transmembrane domain: PDB 6TYI^20^ and periplasmic domain: PDB 2PFU)^19^. Created in part in Biorender.

In Gram-negative bacteria, there are two proposed models^7,24–26^. Both utilise a TonB-dependent transporter (TBDT) for the recognition and transport of the iron-bound siderophore into the periplasmic space. The energy for this transport is provided by the proton motive force transduced through the ExbBD-TonB complex. Once the siderophore complex reaches the periplasmic space, the two models take different paths. In, for example, enterobactin uptake in *E. coli*, the siderophore-iron complex is transported into the cytoplasm through an ATP-binding cassette (ABC) transporter (Fig. 2, Blue pathway)^7^. This process might be aided in the periplasm by a carrier protein. The iron is released in the cytoplasm by enzymatic digestion of the siderophore (Fig. 2)^7^. In the alternative pathway (Fig. 2, Red pathway), iron is liberated from the complex in the periplasmic space via reduction of Fe^III^ to Fe^II^, as siderophores have low affinity for Fe^II^ ^25^. From here the iron is transported into the cytoplasm via an ABC transporter and potentially a carrier protein^25^. The siderophore is then either broken down or recycled by the organism. A typical example of this system is the pyoverdine uptake system in *Pseudomonas aeruginosa*^25^.

The uptake system in Gram-positive organisms is not as well studied^26^. The absence of an outer membrane, means there is no TBDT-TonB-ExbBD system. The most well-established model of uptake is the Iron Shuttle Model (Fig. 2, Green pathway) by Raymond et al. later revised by Wencewicz^27,28^. In this case, there are free siderophore molecules in the extracellular space and bound to the siderophore binding protein (SBP). Some SBPs have been structurally characterised^27,29,30^. In the Raymond system, the extracellular siderophore scavenges iron, which is then passed to the siderophore molecule bound to the SBP^27^. The SBP then closes onto the ABC transporter and lets the siderophore complex through the bacterial membrane^27^. Wencewicz revised this by replacing the extracellular siderophore with transferrin, proposing that the SBP bound siderophore strips iron from transferrin^28^.

### Sideromycins

**Fig. 3:**
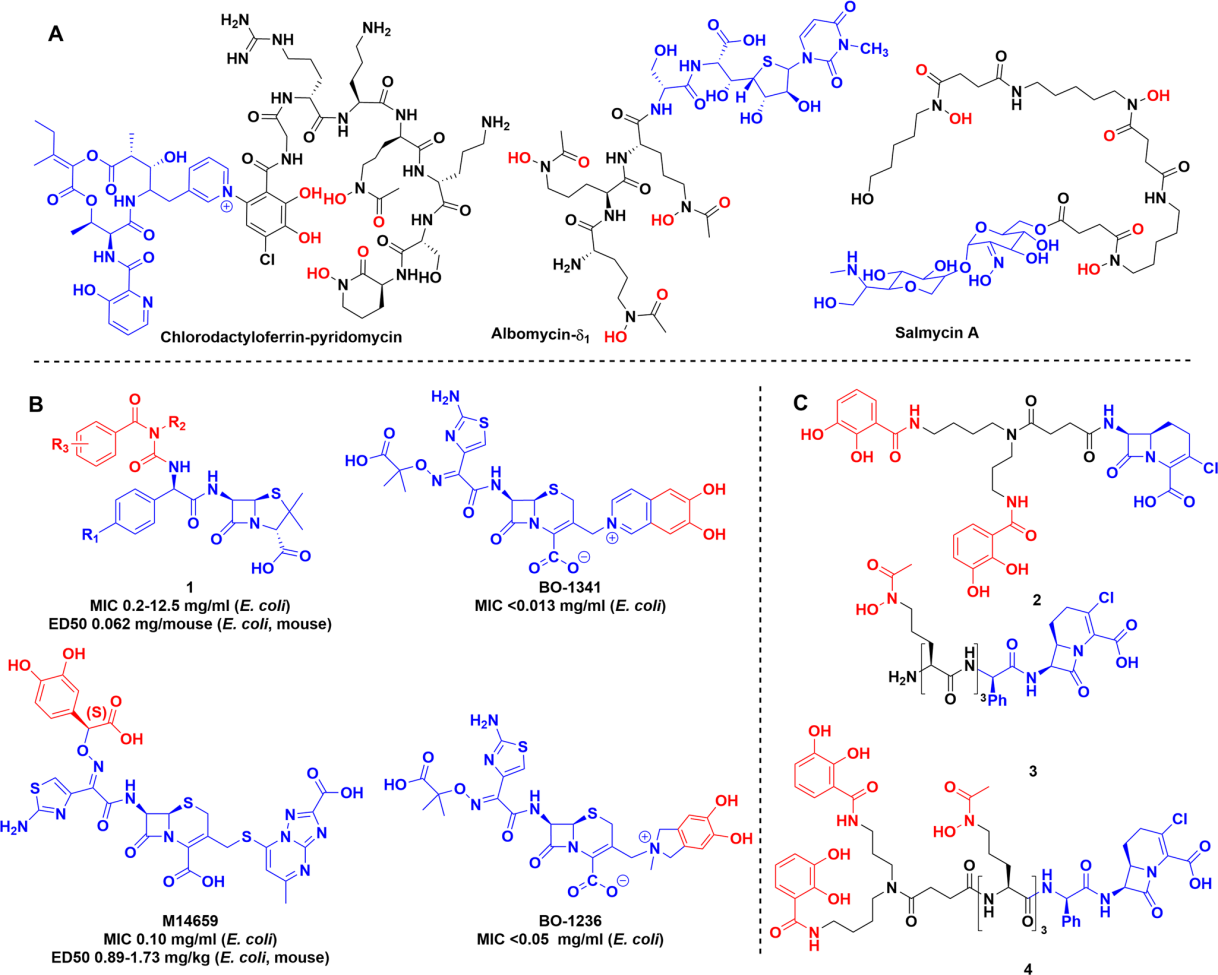
Sideromycins and examples of early siderophore conjugates. **A** Natural product sideromycins. **B** Early siderophore drug conjugates produced by industry. **C** Early synthetic siderophores used to probe the limits and selectivity of bacterial uptake systems. Chemdraw Professional version 22.2.0.3300.

Alongside siderophores, some bacteria have evolved an ingenious Trojan-horse approach utilising their competitors need for iron against them by biosynthesising siderophore-antibiotic conjugate natural products known as sideromycins (Fig. 3A)^9,31^. Albomycin is the best-known sideromycin and naturally combines an iron-chelating moiety to hijack siderophore uptake systems with an antibiotic warhead that is released by peptidase-mediated cleavage of the metal chelator. This releases the seryl- tRNA synthetase inhibitor as an antibiotic^32^. Albomycin has a quite broad activity against both Gram-positive and Gram-negative bacteria^33^. In addition, salmycin, isolated from *Streptomyces violaceus* by Vértesy et al.^34^, was shown to be active against a smaller subset of bacterial strains compared to albomycin, although its mechanism of action is unknown^35,36^. A more recent discovery is the natural product conjugate of chlorodactylloferrin and pyridomycin described by the Hartkoorn lab. The two compounds are separately biosynthesized, directed by a hybrid biosynthetic gene cluster. The final conjugation step is facilitated by the redox active ferric ion. The formal C–N cross coupling is thought to occur through oxidation of the catechol group to the quinone form, followed by attacked by the pyridine in a Michael-type addition reaction^37^. This was confirmed by experimental data using other oxidising agents, redox active and non-active metals^37^.

The fascinating ability of microorganisms to biosynthesise siderophores and sideromycins has inspired many researchers to develop conjugates to hijack the siderophore uptake system for drug delivery. Following decades of research, a Trojan horse-like antibiotic, cefiderocol, was approved for clinical use in 2019. This has given significant credence to the Trojan horse concept and its potential. Here we review the current state of the field, and we note that there is significant promise in the use of such molecules not just as antibiotics but also both in diagnostics and as chemical biology tools to understand fundamental aspects of microbial iron homoeostasis and its role in pathogenicity.

### Trojan horse antibiotics

The strategy of synthetic Trojan horse antibiotics is multifaceted:

- conjugation of a siderophore to an antibiotic enabling greater uptake, reducing the minimum inhibitory concentration (MIC) and thus the effective concentration required for activity;
- improved selectivity, i.e. using the siderophore to target antibiotics to specific pathogens thus avoiding the negative effects of broad-spectrum antibiotics on the patient’s microbiome^38^; and
- modifying attributes of existing molecules either by adding antibiotic activity or bacterial selectivity.

Early work in the field focused on β-lactam antibiotics as the warhead moiety due to the ease of conjugation and their high tolerance to peripheral substitution. The conjugated siderophores were initially simple iron-chelating moieties, e.g. catechols, natural product siderophores or analogues thereof.

Ohi et al. were one of the pioneers in the field, they synthesised a substantial library of catechol conjugated ureidopenicillins, ureidocephalosporins and ureidocephamycins (Fig. 3, **1**)^39–41^. They were tested against multiple bacterial strains, some of which displayed β-lactamase activity, and several compounds in the library showed improved efficacy against multiple Gram-positive (*Staphylococcus aureus* 209P JC-1 and JU-5) and Gram-negative organisms (*E. coli* NIHJ JC-2, *Klebsiella pneumoniae* JU-90, etc.) compared to the parent compound both in vitro and in vivo^39^.

Mochizuki et al. developed a modified cephalosporin, M14659, and studied iron binding and uptake using isotopic labelling (Fig. 3B)^42,43^. Antimicrobial activity tests of M14659 against *E. coli* were also performed in the presence of exogenous chelators, i.e. transferrin, lactoferrin and dipyridyl to reduce bioavailable iron. Adding any of these molecules to the media increased the bactericidal activity of the conjugate at half MIC by 30-fold after 2 h and 6000-fold after 4 h^43^. Using a ^14^C labelled derivative showed that uptake is ATP-dependent and requires low, but measurable levels of extracellular iron. This is supported by the exchange assay between ^59^Fe filled transferrin and M14659, which showed no iron transfer between the two molecules^43^.

Nakagawa et al. designed 6,7-dihydroxy-isoquinolium linked ceftazidime conjugates BO-1341 and BO-1236 resulting in similarly improved efficacy^44^. The modifications did not impact the β-lactamase stability compared to the parent antibiotic (Fig. 3B). Studies of *E. coli* mutants, in which different uptake systems, i.e. *tonB*, (encoding siderophore uptake protein) *envZ*, (encoding porin forming kinase) *ompF*, *ompC* (encoding generic porin forming proteins) were knocked out, showed that the uptake of cephalosporin conjugates BO-1341, BO-1236 occurs through siderophore pathways and not via porins known to be responsible for cephalosporin uptake. This result indicates that the new uptake mechanism appears to be responsible for greater efficacy of the conjugate^45^.

These reports demonstrated an important proof-of-concept, although they noted that better mechanism of action studies were required to shed light on the factors that resulted in improved activity. Did improved uptake result from the siderophore conjugation, or did the catechol group provide improved solubility? Maybe the conjugates utilised a mechanism of action distinct from the parent antibiotic? This work also demonstrates the potential of siderophore conjugates as probes to understand uptake mechanisms.

To further untangle the contributions of different conjugates and investigate bacterial uptake^46–51^, Miller et al. created several Trojan horse derivatives including hydroxamate and catechol β-lactam conjugates **2** and **3** (Fig. 3). MICs were measured against several wild-type and mutant bacterial strains^46–49^, showing that both hydroxamate and catechol based siderophore conjugates were effective at inhibiting early bacterial growth, but mutants lacking the respective uptake proteins FhuA (for uptake of hydroxamate siderophores) and CirA (for uptake of catechol siderophores) became dominant^51^. Cross-resistant bacteria were observed, but they were relatively rare and non-viable in iron-deficient media^50^. In later work, Ghosh et al. used mixed hydroxamate and catechol moieties to generate conjugate **4** with activity against some *Staphylococcus* species, as well as *Klebsiella* and *Escherichia* when mixed with the suicide β-lactamase inhibitor, sulbactam (Fig. 3)^52^. It maintained activity against strains containing multiple *fepA* (enterobactin transporter) and *cirA* mutations.

**Fig. 4:**
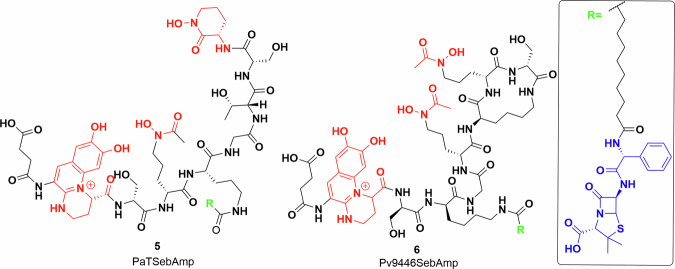
Siderophore conjugates to target *Pseudomonas aeruginosa.* Pyoverdine–ampicillin conjugates semisynthesised by Kinzel et al.^61^. Iron-chelating groups shown in red. Inset: ampicillin (blue) and alkyl linker (black). Chemdraw Professional version 22.2.0.3300.

There has also been significant work towards developing siderophore conjugates to increase the efficacy of existing antibiotics against *P. aeruginosa*, as it is a leading cause of serious lung infection in immunocompromised patients including those suffering from cystic fibrosis^53^. The production of siderophores, pyochelin and pyoverdines has been implicated in virulence of *P. aeruginosa*, leading to study of their production and uptake^54–58^. Intriguingly, three groups of natural pyoverdine analogues are made by different *P. aeruginosa* strains^59^. In general *Pseudomonas* sp produce pyoverdines of one group and thus can utilise siderophores of the same group even if produced by other strains. Meyer et al. labelled the outer membrane receptors responsible for the uptake of pyoverdine with fluorescent antibodies and showed that these are the most abundant iron-regulated outer membrane proteins in *P. aeruginosa*^60^. Budzikiewicz et al. prepared β-lactam conjugates of two pyoverdines, **5**, **6** from two different serotypes of *P. aeruginosa* grown in iron-deficient media (Fig. 4)^61^. The uptake of the conjugates was measured against the same and other serotypes, determining the MIC and growth profiles. The results fit into the delayed growth trend reported by the Miller lab. *P. aeruginosa* is inherently ampicillin resistant, but the ampicillin–siderophore conjugates showed an MIC around 0.04 and 0.67 μg/ml. However, after a day, a different serotype of *P. aeruginosa* started growing in the media, which was resistant to the applied siderophore ampicillin conjugate^61^.

These studies showed that the recognition of a siderophore is mostly dependant on the chelating functional group, however, it did not address more subtle issues, such as the steric tolerance of the transporter, or if attaching a siderophore changes the ability of the antibiotic to bind to its target. There are significant limitations to our understanding of siderophore uptake systems at the molecular level. Greater insight is needed to enable rational design of effective antibiotic-siderophore conjugates. However, as illustrated above, siderophore conjugates can be used as chemical biology tools to probe siderophore uptake systems to inform structure-activity relationships.

### Rational design of siderophore conjugates

During the early work by Miller, Budzikiewicz and several pharmaceutical research labs as described above, it became obvious that multiple factors need to be considered in conjugate design. Since then, there has been extensive research into siderophore uptake transporters, although, this has been complicated due to the challenges associated with characterising membrane-bound proteins and multicomponent systems. Thus, studies have included understanding siderophore recognition patterns, uptake tolerance to siderophore modifications, ability of the transporter to accommodate larger molecules, charge limits, etc. In addition, how siderophores are recognised by the immune system must be considered. For example, catecholate siderophores such as enterobactin, or catecholate-like siderophores such as carboxymycobactin are recognised by the immune protein, siderocalin that intercepts and binds bacterial siderophores thus helping to control infection. However, hydroxamate siderophores are not recognised by siderocalin^62–64^. In the next section, we examine the considerations in the design of siderophore conjugates.

#### Choice of siderophore and transport system

**Fig. 5:**
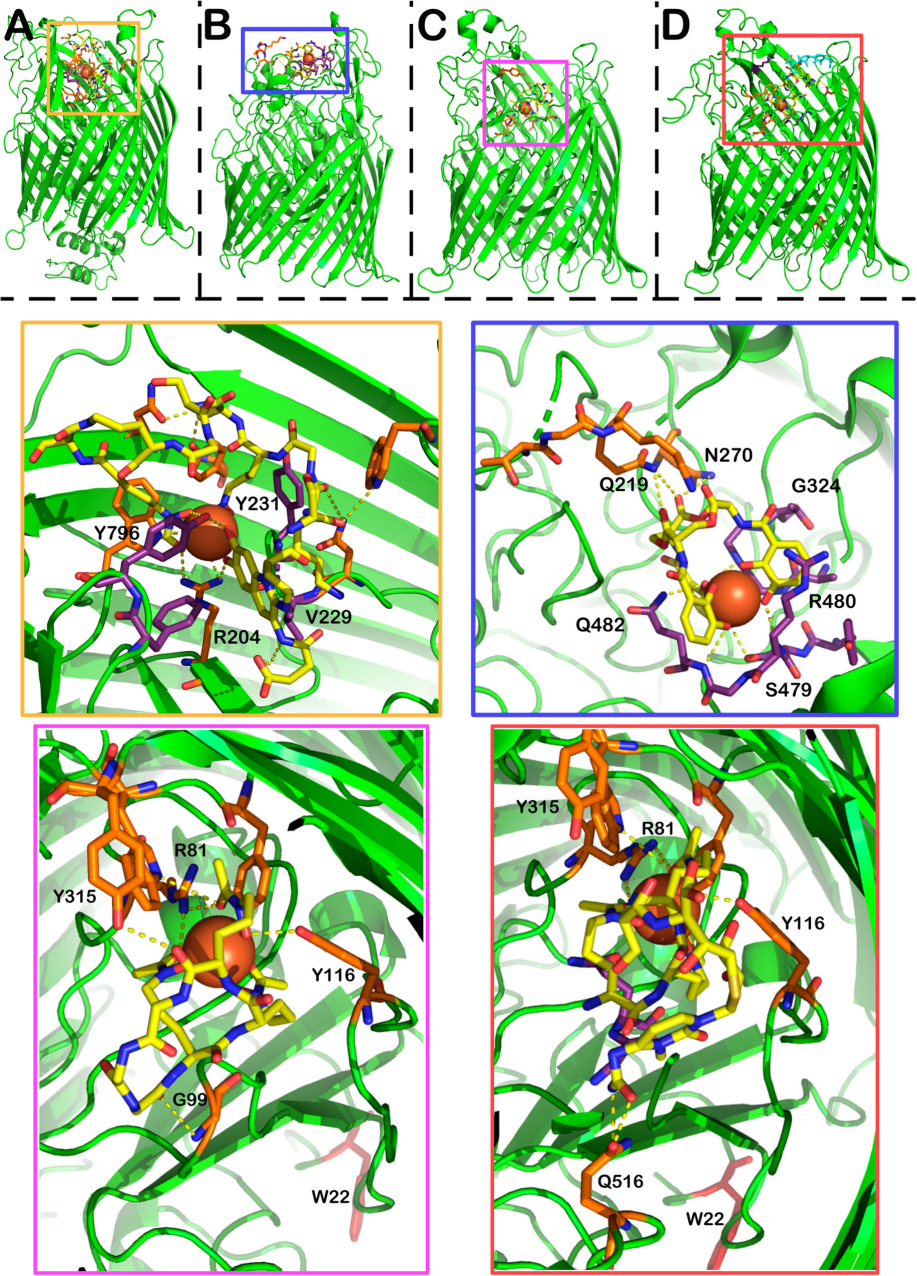
Ligand binding modes to siderophore outer-membrane transporters. **A** X-ray crystal structure of FpvA bound to pyoverdine (PDB 2W6T) (Chain A omitted for clarity). **B** X-ray crystal structure of PfeA (PDB 6Q5E) bound to enterobactin **C** X-ray crystal structure of FhuA bound to ferrichrome (PDB 1BY5) **D** X-ray crystal structure of FhuA bound to δ2-albomycin (PDB 1QKC). The structures show both two albomycin conformers in yellow and cyan. **Yellow Zoom**: binding pocket of FpvA with pyoverdine in yellow, ferric ion represented by a sphere. The orange amino acid residues correspond to hydrogen bonding interactions between the siderophore and the uptake protein, while the purple residues provide mostly alternative bonding interactions. **Blue zoom**: enterobactin binding pocket with interactions shown using the same colouring scheme. **Pink zoom**: Ferrichrome binding pocket with interactions shown. **Orange zoom**: albomycin binding pocket with interactions shown, the image picture omits the cyan conformer for clarity.

The first step is the identification of the uptake pathways of different siderophore classes which can be coupled with understanding of their selectivity. Here we focus on Gram-negative systems due to their importance to AMR. Initial insight into sideophore uptake was gained through siderophore ^55^Fe labelling and knock-out studies of outer membrane proteins of producing strains^65–67^. However, this just confirms that the metal has been taken up by the bacterium and not whether the siderophore itself has been internalised^27^. The multicomponent nature of these systems, with components localised in outer and inner membranes in Gram-negative bacteria, has presented significant challenges, however, several structural studies have revealed important insight into substrate–protein interactions. As mentioned above, siderophore biosynthetic genes are usually clustered with dedicated membrane-bound uptake transporters, several of which have been structurally characterised including the enterobactin transporter FepA, pyoverdine transporter FpvA and ferrichrome transporter FhuA (Fig. 5).

FepA is responsible for ferric enterobactin transport into *E. coli* cells^68^. FpvA fulfils the same role in *P. aeruginosa*, transporting ferric pyoverdine^69^. Ferric siderophore transport proteins share a similar tertiary structure, a membrane spanning beta barrel, a turn-rich internal plug and finally an unstructured connecting region in the periplasm^24^. Early fluorescent and spin labelling studies established a two-step process of ligand binding and then internalisation^70,71^. This was supported by the crystal structures of FepA (PDB 1FEP) and the co-crystallisation of ferric-enterobactin and PfeA (PDB 6Q5E) (Fig. 5B), a FepA homologue in *Pseudomonas*^17,72^. Moynie et al. identified two binding sites for ferric enterobactin in PfeA^72^. The structure of these sites appears to be highly complementary to the 120° gaps between the catechol groups, with extensive hydrogen bonding and cation–pi interactions with the rings^72^. The most important interactions appear to be R480 forming an electrostatic/cation–π interaction and Q428 on the other side of the catechol ring, and the backbone of the G324 and G325 residues wedging themselves into the remaining space^72^. The catechol rings form hydrogen bonds to the backbone nitrogens of R480, G325 and Q482 and side chains of S479 and Q482^72^. The trilactone ring is supported by a hydrogen bond between two of the ester groups and Q219^72^. Intriguingly, co-crystallisation studies also showed FepA binding to azotochelin as well as protochelin, indicating the promiscuity of the transporter^72^. The two smaller siderophores occupied the same binding sites, maintaining similar binding interactions^72^.

The binding of pyoverdine to the transporter FpvA has been elucidated in crystallographic studies by Greenwald et al. (Fig. 5A)^73^. Importantly, they recognised that only one of the molecules in the two-part FpvA asymmetric unit can bind pyoverdine due to a steric clash between the second molecule and the neighbouring asymmetric unit (PDB 2W6T). Key interactions include V229 and Y231 in contact with the chromophore and the nearby hydroxamate. Intriguingly, R204 moves more than 8 angstroms from its position in substrate-free form to place its sidechain into binding distance with the iron and the catechol unit, illustrating the flexibility and dynamic nature of these sites.

FhuA is the receptor responsible for the uptake of ferrichrome-bound iron in most bacteria and one of the first to be structurally characterised^74,75^. The overall structure of the receptor resembles FepA and FpvA with the two easily identifiable domains (beta barrel membrane spanning region and internal plug) (Fig. 5C, D). The binding domain of ferrichrome is lined with aromatic residues, forming a pocket, R81 H-bonds with two hydroxamate carbonyls. The third hydroxamate carbonyl is in hydrogen bonding distance with Y244, while one of the residues (Y116) can bond with the OH group of the hydroxamate. The macrocycle of ferrichrome is bound by G99 and Y315, however, it is still accessible to the solvent, while the iron-bound side is buried in the transporter. This seems to be an important factor, because the R81 residue shows large movement between the ligand free and the bound state. The move seems to propagate an asymmetric unfolding of the helical structure on one side of the plug domain, forming a tunnel for the transport of ferrichrome into the periplasm. In the closed state, this tunnel is blocked by W22 which moves ~17 Å between the closed and open states.

FhuA bound to the sideromycin, albomycin, has also been characterised giving an excellent opportunity to compare the structure of a siderophore conjugate to the native siderophore bound structure, allowing for a greater understanding of how the larger conjugates are accommodated (Fig. 5)^76^. Generally speaking, the residues binding the hydroxamate groups (R81, Y116, Y244) remain in place. The major differences between the structures are in the hydrogen bonding of the backbone of the molecule with the protein. Y315 loses its binding partner while G99 only provides weak van der Waals contact. There are multiple new hydrogen bonds, depending on the conformation of the albomycin molecule. In the more compact state of albomycin, Q516 forms hydrogen bonds with the amide moiety at the end of the albomycin payload.

The interactions described above indicate that the iron-binding moieties of siderophores appear to be the most important points of interaction with the receptor and may explain the promiscuity of some receptors. It is reasonable to suggest that the electrostatic interactions surrounding the ferric ion are more important for the recognition of the molecule. This of course would represent a benefit to the organism. The ability to utilise not just its own siderophores but those produced by competing pathogenic or commensal bacterial species enables strain survival^16,77,78^.

#### Functionalisation/modification tolerance

Insight into siderophore–protein interactions in relation to recognition and uptake should enable rational design of conjugates. Thus, the structural studies of uptake receptors are complemented by feeding experiments in bacteria using libraries of siderophore conjugates, illustrating the tolerance of uptake systems to modifications of the siderophore and the payload.

**Fig. 6:**
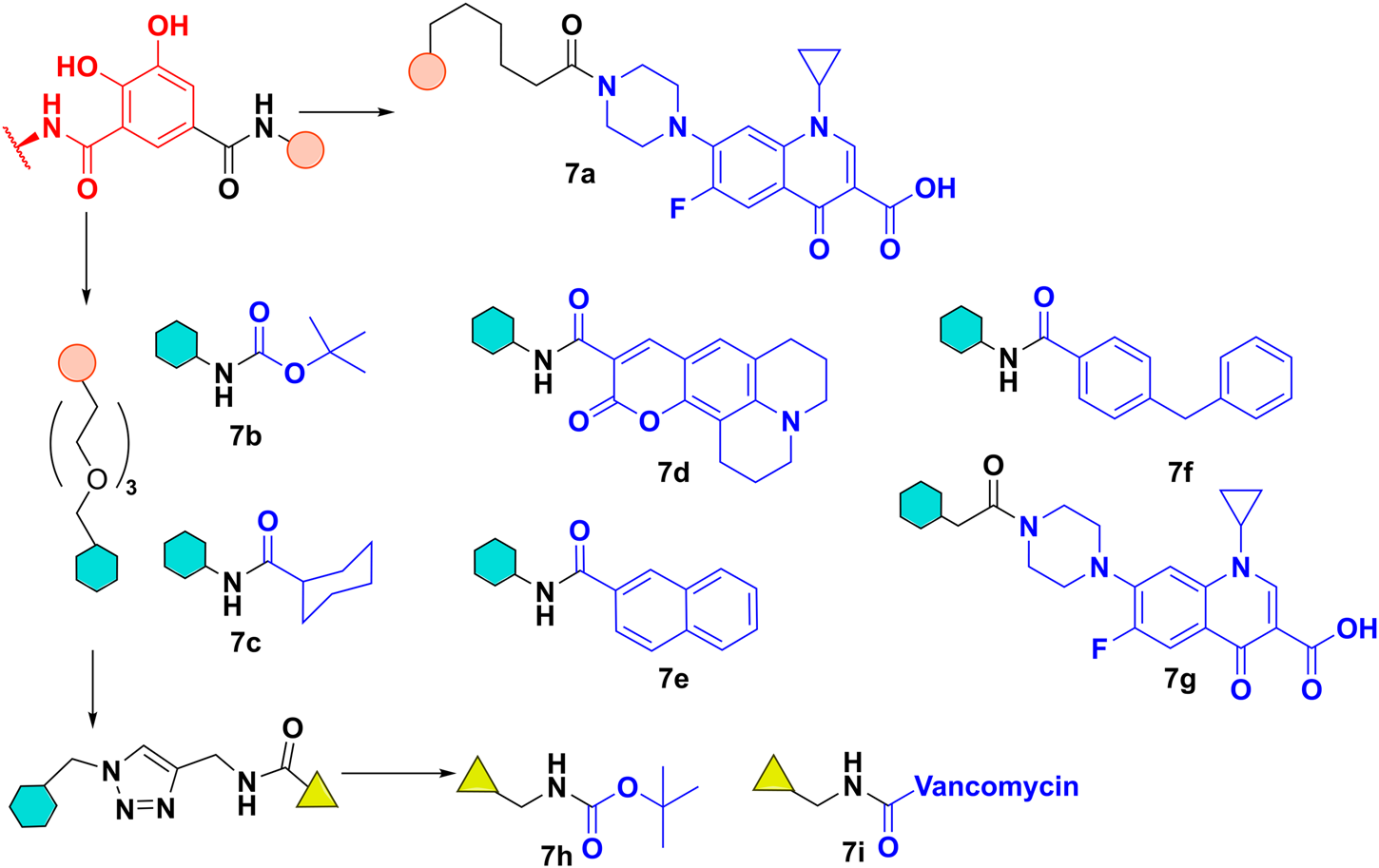
Combinatorial siderophore-antibiotic conjugate library. Work by Nolan et al. The shapes correspond to the connection point along which the different building blocks can be assembled. Chelator—red; payload—blue. Chemdraw Professional version 22.2.0.3300.

Nolan et al. synthesised a variety of substituted enterobactin derivatives with various groups attached to PEG linkers (Fig. 6)^79^. Using growth recovery experiments, they concluded, unsurprisingly perhaps, that in both *E. coli* and *P. aeruginosa*, there is a limit to the size of molecule the receptors can accommodate. Knowing the steric limits of these systems is vital information for further design. The *E. coli* receptor (FepA) appears to be selective for smaller cargo, while PfeA (*P. aeruginosa*) can accommodate larger molecules^79^. Further research to compare strains of the same organism used β-lactam antibiotics (ampicillin, amoxicillin) as payloads and measured the activity of the conjugate against unconjugated antibiotics^80^. It was found that even between strains of the same organism there were some differences in growth inhibition, but the differences were not significant when compared to the parent antibiotic.

Comparing their effect on different bacterial species, they found that the conjugate’s activity is highly dependent on the uptake mechanism and the parent antibiotic. Against the β-lactamase producing *K. pneumoniae* ATCC 13883, the conjugates only showed activity when supplemented with the β-lactamase inhibitor sulbactam. *P. aeruginosa* PA01 was completely insensitive, together with *Bacillus cereus* and *S. aureus*. Earlier research from the same group showed uptake in *P. aeruginosa*, which supports the argument that the strain is inherently insensitive to the payload^79^. Other groups had similar findings in terms of payload size. Zscherp et al. made multiple fluorescent conjugates and their uptake in *E. coli* and *P. aeruginosa* were similar to the molecules tested by the Nolan group^81^. Comparing the payloads reported, it seems a balance of size and flexibility are significant factors in uptake, however, it is not easy to define hard spatial or structural rules regarding the activity of new and untested conjugates.

#### Chirality

**Fig. 7:**
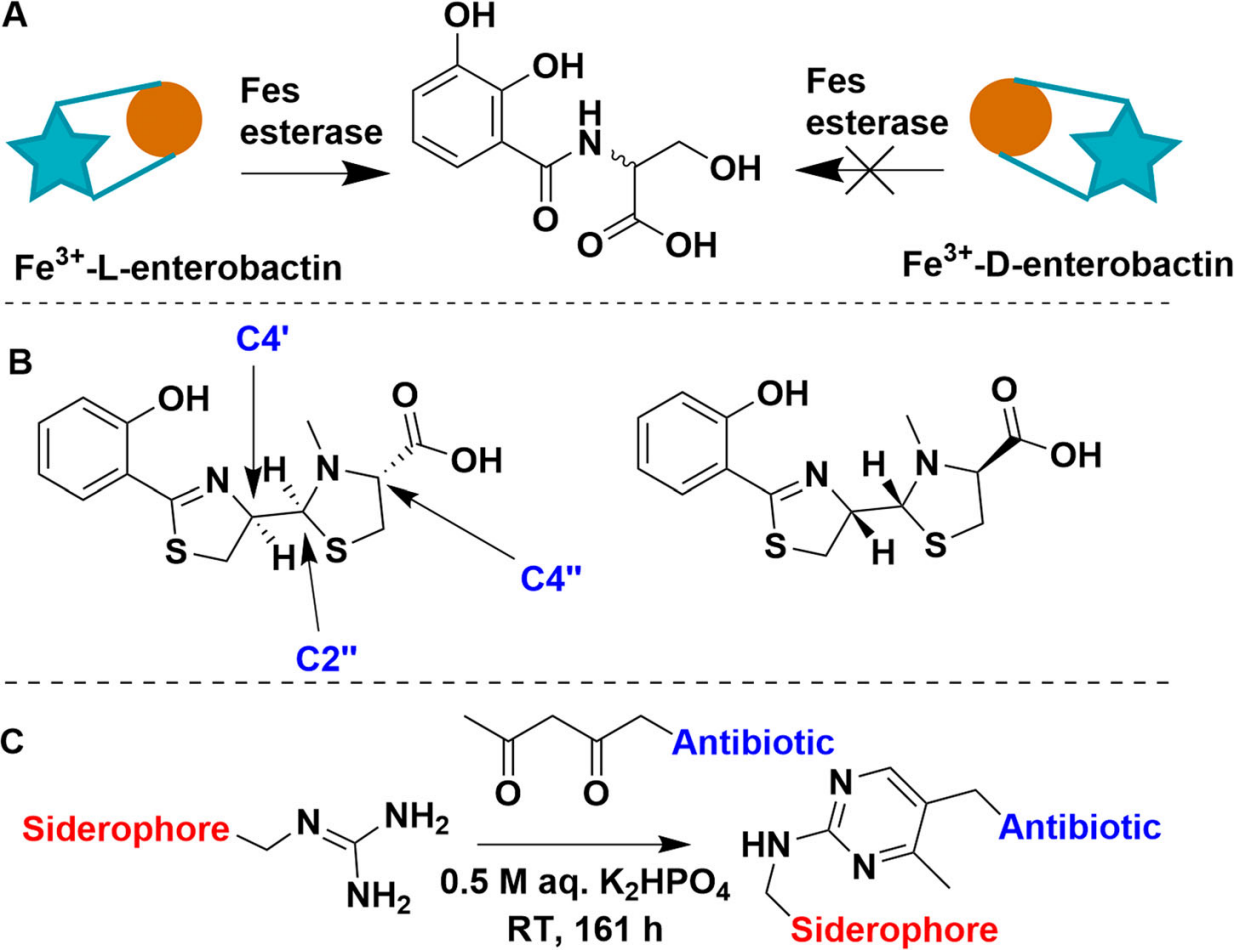
Siderophore stereochemistry. **A** Schematic representation of the stereoselectivity of Fes esterase. **B** The structure and chiral centres of pyochelin on the left and enantio-pyochelin on the right. **C** Arginine and beta-diketone condensation forming a pyrimidine linker. Chemdraw Professional version 22.2.0.3300.

One would wonder, does the stereochemistry of the siderophore matter? This is of course relevant to natural siderophores, most of which are produced as single stereoisomers, and it would thus be reasonable to assume that transporters are stereoselective. The answer however is more complex. Enterobactin and its cognate receptor in *E. coli*, FepA, cannot differentiate between l- and d-enterobactin^79,80^. However, the internal esterase (Fes), responsible for liberating the iron from enterobactin, solely recognises the l variant (Fig. 7A)^82^. Thus d-enterobactin becomes a useful Trojan horse starting point, because using synthetic d-enterobactin enables access to the cytosol while denying the bacterial cell access to the chelated iron^80,83,84^. The picture is very different for other siderophores. Next to pyoverdine, pyochelin is one of the native siderophores of *P. aeruginosa*, while enantio-pyochelin is produced by *Pseudomonas fluorescens* (Fig. 7B)^85^. While the two uptake systems, FptA (pyochelin) and FetA (enantio-pyochelin), share a high structural homology, they have very low sequence identity (25.1%)^86^. Indeed, they create entirely different binding pockets, thus making the siderophore pair incompatible with each other^86^.

#### Linkers and attachment points

Initial work on conjugate synthesis utilised bioconjugation anchors on the siderophores (carboxylates, amines) to attach payloads directly or via alkyl chain linkers^46,50,52,61^. This works relatively well for β-lactam antibiotics due to their tolerance to peripheral modifications but proved limiting of other payloads, as siderophores have limited natural conjugation sites, even when taking some unorthodox bioconjugation strategies into consideration. For example, Kinzel and Budzikiewicz utilised a beta-diketone and arginine sidechain condensation to generate a pyrimidine-linked pyoverdine D and β-lactam antibiotic. The resulting conjugate facilitated iron uptake but was unfortunately ineffective as an antibiotic (Fig. 7C)^87^.

Frequently when siderophores are conjugated to glycopeptides^88^, macrolides^89^, or fluoroquinolones^90–94^ using a covalent alkyl chain linker, the antibiotic activity of the conjugate is reduced or eliminated compared to the parent antibiotic. This led to investigation into the importance of linker length and the use of biologically labile linkers (e.g. those cleaved via endogenous esterases or other enzymes). Out of the alternative antibiotics, nor- and ciprofloxacin are the most regularly utilised as payloads due to the ease of conjugation through the secondary amine.

**Fig. 8:**
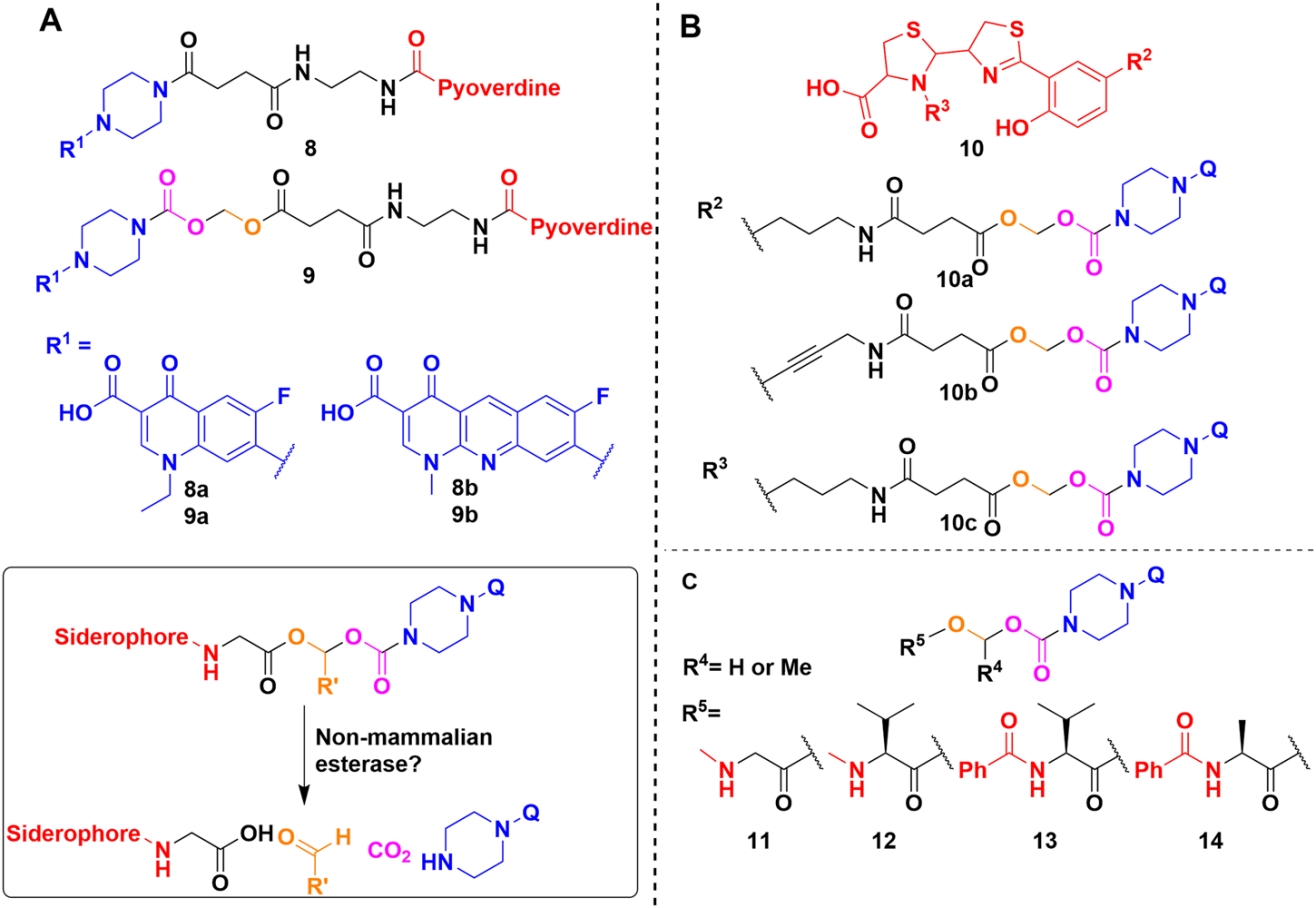
Siderophore linker and conjugation strategies. **A** Pyoverdine conjugated to norfloxacin (**8a**, **9a**) and to benzonaphthyridone (**8b**, **9b**) using a hydrolytically stable or labile linker. Inset—products of the linker cleavage reaction. **B** Pyochelin conjugates using the same quinolones as the pyoverdine experiments by Herrard et al., probing linker flexibility and conjugation points. **C** Library of linkers used by Zheng and Nolan. Q represents a quinolone. Chemdraw Professional version 22.2.0.3300.

For example, Herard et al. synthesised a small library of conjugates using pyoverdine isolated from *P. aeruginosa* ATCC 15692 (Fig. 8A)^95^. Bioassays of **8a**, **8b**, **9a**, **9b** found that the hydrolytically labile linkers result in somewhat more active conjugates (MIC 1 vs 8 μg/mL). Indeed, the labile-linked pyoverdine-benzonaphthyridone adduct showed better results than the parent antibiotic (MIC 1 vs 16 μg/mL). This makes sense, as pyoverdine uptake stops at the periplasm^25^ and any quinolone conjugate with a stable linker would thus be inactive as it would not reach the cytoplasm. In growth curve experiments the results were similar, with the exception that the labile norfloxacin adduct showed slightly better activity than the parent antibiotic and not the benzonaphthyridone conjugates. However, this was reversed when the adduct was pre-saturated with iron. The fact that conjugates with labile linkers were not substantially more active than the parent compound, also indicated that either the cleavage occurred outside the cell, or that the active uptake and internal cleavage of pyoverdine-conjugate was slower when compared to porin-mediated uptake of the parent antibiotic. Similar results were obtained with pyochelin (Fig. 8B)^91,96^. Molecular docking and inhibitor studies indicated that the pyochelin derivative does interact with the outer membrane protein^96^. However, the lower antibacterial activity compared to the parent antibiotic is again thought to be due to hydrolysis of the linker in the media. This reasoning was supported by an extensive hydrolysis study on (acyloxy)alkyl ester linkers by Zheng and Nolan to establish linker half-life (Fig. 8C)^97^. They generated valuable insight on how the usual CLSI guidelines on end-point antimicrobial activity assays are not suitable for hydrolytically labile linkers^97^. The development of these linkers is of particular interest because fluoroquinolones have been shown to be inactivated when modified at the secondary amine^98^. Based on molecular modelling, even adding 1 or 2 atom linkers inhibits the DNA gyrase binding activity of fluoroquinolones^98^.

**Fig. 9:**
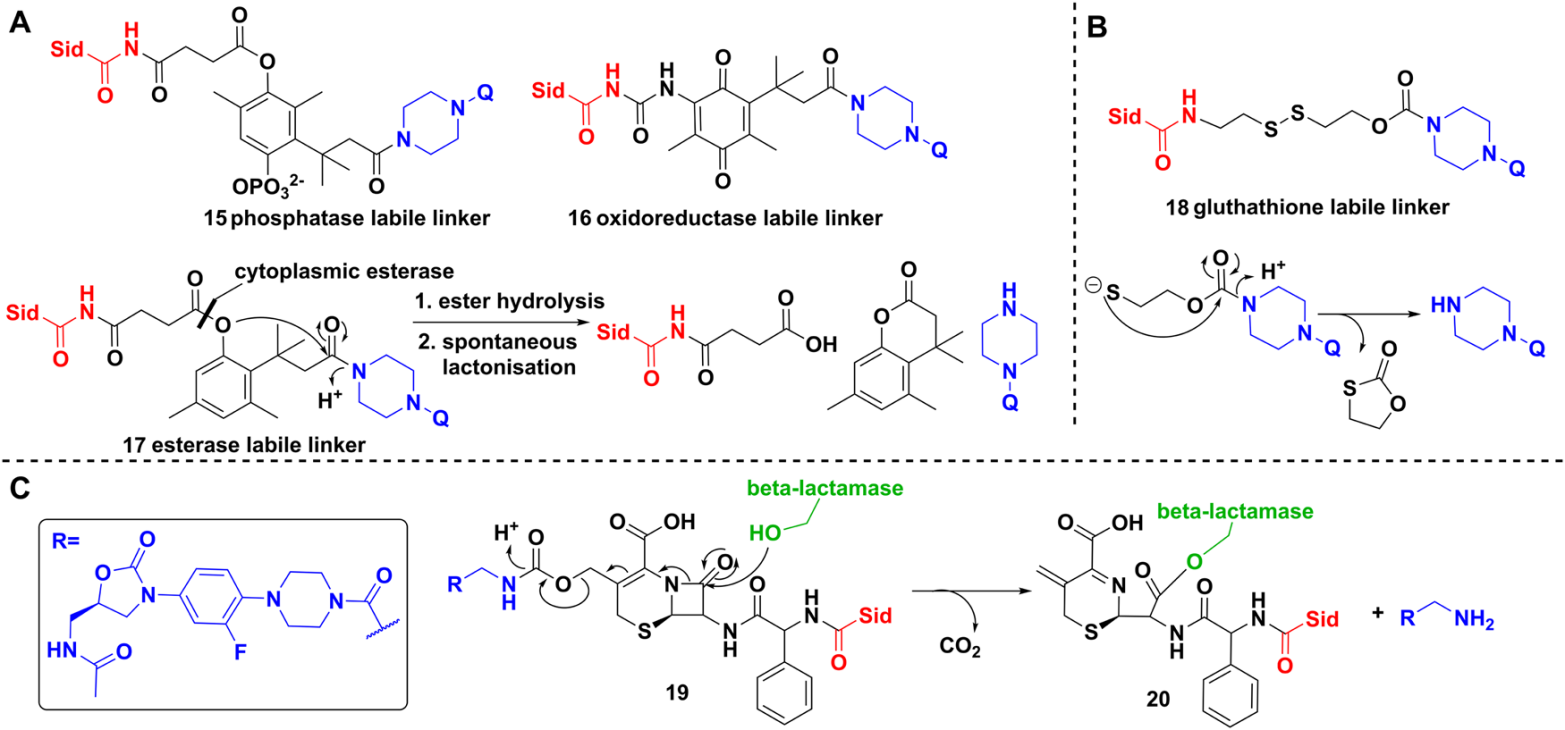
Siderophore conjugation strategies exploiting bacterial biochemistry. **A** Trimethyl locks synthesised by Miller et al. Q represents a quinolone. **B** Glutathione initiated disulfide cleavage and oxothiolanone cyclisation. Q represents a quinolone. **C** Reaction scheme of the β-lactamase initiated ring opening cascade culminating in the release of the oxazolidinone (inset). Chemdraw Professional version 22.2.0.3300.

Other labile linkers such as the “trimethyl-lock” based lactonization have been used in multiple esterase and phosphatase activated prodrug strategies^99–101^. Ji and Miller applied this method to desferrioxamine B–ciprofloxacin conjugates, however, the MIC values showed at least a 32-fold increase compared to the parent drug, which implies either minimal conjugate uptake or poor esterase activity (Fig. 9A)^102^. The phosphatase labile linker **15** showed no activity. Further study of reductase activated linker **16**, gave similar results^103^. Like the (acyloxy)alkyl linkers, the poor activity of these conjugates may be due to extracellular cleavage. The use of thiol-maleimide linkers by Miller et al. resulted in markedly better MICs than the parent antibiotic, indicating siderophore-mediated uptake^104^. However, the in vivo use of these linkers might be hindered by the retro-Michael reaction reported extensively in connection with antibody–drug conjugates^105,106^. Neumann and Nolan evaluated disulfide bond linkages in an enterobactin–ciprofloxacin conjugate^107^. They rationalised that following reduction of the linker by glutathione, the remaining half-linker would collapse into a five membered oxothiolanone, releasing the antibiotic warhead (Fig. 9B). This was successful in vitro, but unfortunately bacterial uptake studies showed lower activity than the parent antibiotic.

Finally, probably the most mechanistically intricate example is a cephalosporin (cephaloglycin) linker (Fig. 9C)^108^. The β-lactam core connected on one side to an oxazolidinone, a ribosomal inhibitor active against Gram-positives, and on the other side to a dicatechol siderophore mimic. The intriguing method of release relies on hydrolysis of the core by bacterial β-lactamases, e.g. serine β-lactamase (ADC-1). Theoretically, metallo-β-lactamases would produce the same effect. The conjugates had good activity against *Acinetobacter baumannii*, *E. coli* and *P. aeruginosa*, either by the activation and release of the oxazolidinone, or by the inherent antibacterial activity of the cephalosporin^108^.

#### Payloads

In much of the work on siderophore conjugates, as described above, the most well-established payloads are existing antibiotics. Quinolones and β-lactam antibiotics are readily available and mostly non-toxic to humans, thus making them an obvious payload choice. However, the ability of siderophores to shuttle molecules through the bacterial membrane is an exciting opportunity to investigate compounds with either no preexisting antibacterial activity due to uptake issues, or molecules that display low selectivity for bacterial over mammalian cells.

**Fig. 10:**
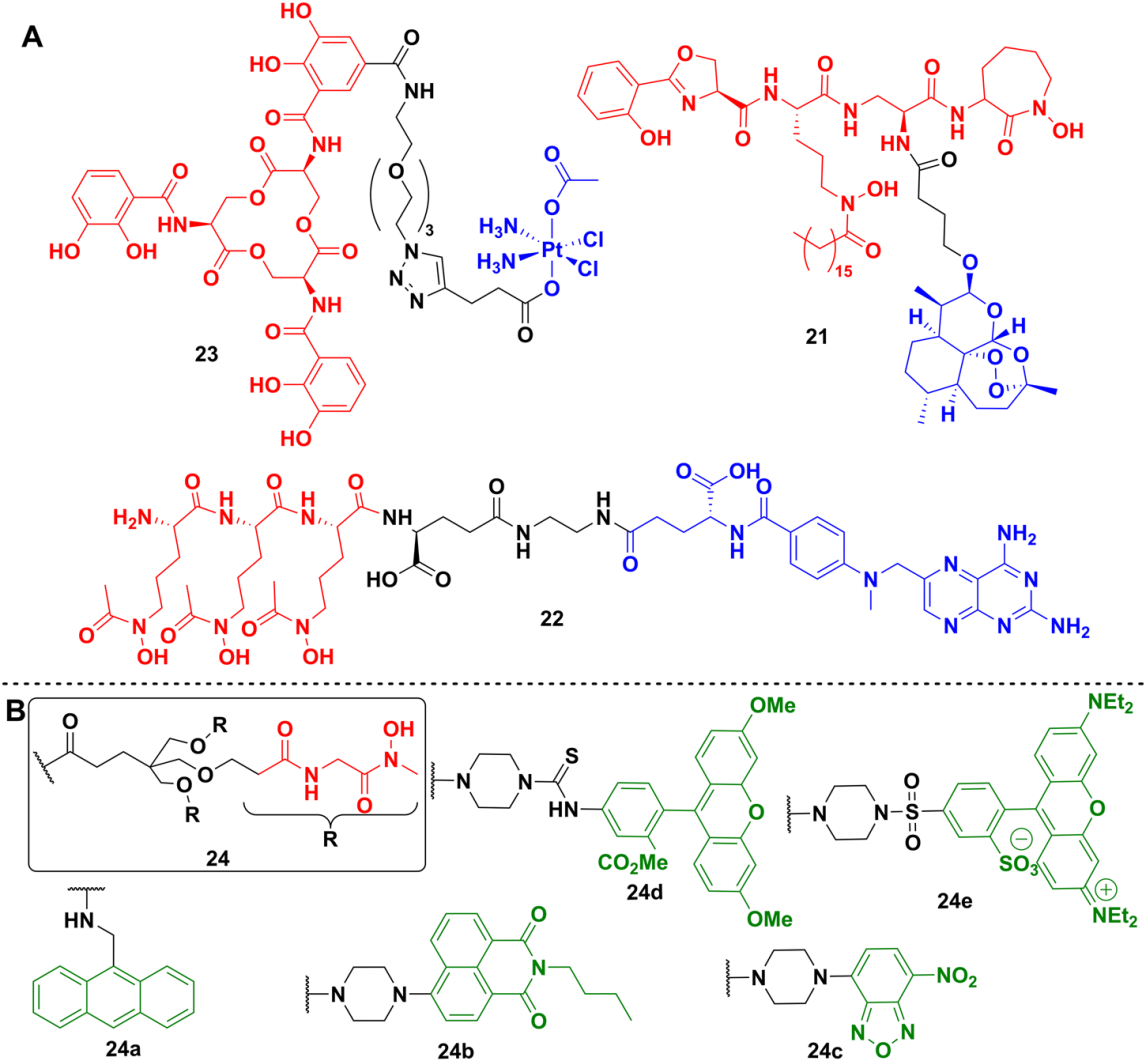
Repurposed and fluorescent siderophore conjugate payloads. **A** Repurposed payloads. Enterobactin–cisplatin conjugate **23**. Mycobactin T analogue conjugated to artemisinin **21**. The most promising methotrexate conjugate **22** synthesised by Zhao et al. (siderophores—red; linkers—black; payload—blue). **B** Multiple fluorescent probes used to label a biomimetic desferrichrome analogue. Chemdraw Professional version 22.2.0.3300.

The first repurposed warhead reported was artemisinin by Miller et al. (Fig. 10)^109^. The antimalarial drug artemisinin (IC_50_ < 0.0036 μg/ml) is a sesquiterpene natural product with an unusual transannular 1,2,4-trioxane group^109^. The mechanism of action of the molecule is based on the homolytic cleavage of the trioxane by ferrous ions forming highly active radicals^110^. Artemisinin is inactive against bacteria because it cannot cross the cell membrane, however, when linked to a mycobactin T analogue **21**, it shows remarkable activity and selectivity towards *Mycobacterium tuberculosis*^109^. The conjugate’s MIC against multiple MDR and XDR strains is below 1.25 μg/ml, and against fast-growing *Mycobacteria* and Gram-negative and -positive species it is > 12.5 μg/mL^109^.

Anticancer medications are also a good source of potential warheads due to their ability to damage DNA or inhibit DNA synthesis, although cytotoxicity and selectivity are potential issues. Zhao et al. generated conjugate **22** from methotrexate and a trihydroxamate siderophore derived from albomycin (Figs. 3, 10)^111^. Methotrexate is a dihydrofolate reductase inhibitor but has poor penetration of bacterial membranes. The most active conjugate had an MIC against Gram-positive *Streptococcus pneumoniae* of 1.72 nM and Gram-negative *Y. enterocolitica* of 6.8 nM. No activity was observed against *Staphylococcus epidermidis, Salmonella enterica* serovar *Typhimurium* and *A. baumannii*. It is important to note, that off-target drug toxicity is of great concern when repurposing anticancer drugs. The methotrexate conjugates showed a large decrease of toxicity (around 2000-fold) against human liver cell line L02, which coupled to the increased antibacterial activity makes it a promising approach.

The Nolan lab also described repurposing the DNA crosslinking, antitumour drug, cisplatin, as a Trojan horse payload linked to enterobactin **23** (Fig. 10)^83^. The mechanism of action of the drug did not cause complete killing of bacterial cells on agar, although remaining cells were not viable. The conjugate resulted in ~50% decrease in the number of viable cells at 60 μM. Knocking out the outer membrane transporter (*fepA*) and the inner membrane protein complex (*fepCDG*) independently, significantly inhibited conjugate uptake. However, changing the stereochemistry of the enterobactin moiety in the conjugate from the l enantiomer to the d increased its activity against *E. coli*. This was explained as resulting from the inability of the Fes esterase to act on the d enantiomer. In a follow-up study, oxaliplatin was used as the platinum-based payload but that proved to be less effective^112^. Investigating the DNA damage caused by the conjugates using a *lacZ* reporter coupled to the SOS response of the bacterium showed lower and later activity with the oxaliplatin drugs, compared to cisplatin^112^.

While most payloads utilised are usually small molecules, this is not necessarily a limitation on the uptake systems. The Schalk and Brönstrup labs coupled synthetic siderophore mimics to synthetic TonB-box peptide fragments^113^. The underlying idea is that after uptake, the peptide fragments disturb the interaction between the outer membrane receptors and the TonB-ExbBD complex. The MECAM conjugates proved to be inactive, while the DOTAM conjugates showed moderate results with some compounds MICs between 0.1 and 4 µM against gentamicin (1 µM). The authors suggest the MICs achieved are an underestimation of the compounds’ activity, as the iron restricted conditions would leave most conjugates in the *apo* form. Overtime conjugate efficiency decreased due to resistance or peptidase action on the peptides. Regardless, this indicates recent groundbreaking work in targeting the siderophore uptake/processing proteins inside bacteria.

Similarly exciting work by Pals et al. explored the use of antisense oligomers (ASO) conjugated to catecholate siderophore mimics to disturb the translation of mRNA into proteins^114^. They chose *acpP* (acyl carrying protein) as their target, due to its crucial role in fatty acid biosynthesis and showed reasonable growth inhibition of *E. coli* in iron rich and poor media. Of the ASOs used, phosphorodiamidate morpholino oligomers showed slightly better results (MIC 0.8 µM) compared to peptide nucleic acids (1.6 µM). Resistance appears to occur via a single-point mutation in the *ybiX* gene, a putative iron-uptake factor and on the same operon as *fiu*, a catechol uptake receptor^114^. Further data provided for *A. baumannii* and human toxicity studies are very promising for in vivo experiments.

### Siderophores as tools for imaging

The selectivity of bacteria for certain siderophores could be a useful attribute in medical diagnostics. For example, using the biosynthetic/transport proteins as biological markers of pathogenic strains or using labelled siderophores to identify the species or phenotype of a certain unknown bacterial sample. This is of particular importance due to the lack of point of care diagnostics for distinguishing bacterial and viral infection as well as distinguishing Gram-negative and Gram-positive bacteria. More accurate profiling would reduce the use of broad-spectrum antibiotics and enable better antibiotic stewardship.

#### Fluorescently labelled siderophores

Fluorescent payloads are not as broadly used as antibiotics to probe siderophore uptake because it is difficult to differentiate between total internalisation and membrane association. Weizman et al. used an anthracene labelled ferrichrome analogue **24a** to probe iron uptake in *Pseudomonas* species (Fig. 10B)^115^. In a later study, radioactive ^55^Fe was used to determine cellular iron uptake and the spectrum of fluorescent molecules was extended to rhodamine, fluorescein and nitrobenzoxadiazole derivatives **24c–e** (Fig. 10B)^116^. While fluorescent labelling has not been extensively used for siderophore conjugate characterisation, alternative uses for fluorescent conjugates have emerged. Hannauer et al. synthesised a biomimetic analogue of desferrichrome linked to a naphtalimide fluorescent reporter called RL-1194, **24b** (Fig. 10B)^117^. They showed that after iron chelation, the compound goes through fluorescent quenching, which is reversible on loss of iron. They used this to follow the recycling of the siderophore and the uptake of iron into *P. aeruginosa*, supporting the argument that some siderophores are recycled and not broken down after entering the cell (Fig. 2)^117^.

Enterobactin, chemoenzymatically modified with an azide, thiol or bromide containing glucose residue, providing a handle for bioconjugation, was leveraged in the chemical synthesis of rhodamine and dansyl labelled monoglycosylated enterobactin conjugates^118^. The Wang group showed that the resulting conjugates were reasonably selective as they labelled multiple strains of *E. coli, P. aeruginosa* and *Vibrio cholerae*, but not *Bacillus subtilis* or *S. aureus*, as these Gram-positive bacteria are not known to use enterobactin. The same group also labelled the citric acid based, *E. coli* siderophore, aerobactin using a fluorescent DIBO derivative^119^. This promising conjugation strategy, while only viable on the siderophore-Fe complex, resulted in successful labelling of *E. coli, K. pneumonia* and *S. enterica*, but not *V. cholerae, S. epidermidis* or *B. subtilis*. However, *S. aureus* and *P. aeruginosa* proved problematic due to auto-fluorescence, highlighting an important consideration for fluorescence detection in whole cells.

Wang et al. also demonstrated the potential for this approach as a diagnostic tool for antibacterial-resistant infection. A dual system of fluorescent probes enabled differentiation of Gram-negative bacteria, vancomycin-sensitive and resistant *Staphylococcus* species^120^. A vancomycin-based probe with Cy5.5 fluorescent head and staphyloferrin A—fluorescein conjugate proved to be a good combination for detecting vancomycin-resistant *S. aureus* in the presence of other bacterial genera^120^.

#### Radiolabelled siderophores for PET imaging

Siderophores can chelate other metals, albeit with lower affinity than iron, and this trait has been exploited for radioactivity based in vivo imaging. Much of the early work focused on ^67^Ga and ^111^In^121–124^. The use of these metal–ligand combinations was limited, however, both radioisotopes with different chelators became important for SPECT imaging^125^. Zirconium was utilised in the early 1990s, however, the targeting moieties used are usually antibodies with a desferrioxamine chelator^126,127^. The other isotopes, ^14^C, ^55^Fe and ^59^Fe, discussed in previous sections as probes for siderophore uptake, are not appropriate for medical diagnostics, due to their long half-life^128^.

**Fig. 11:**
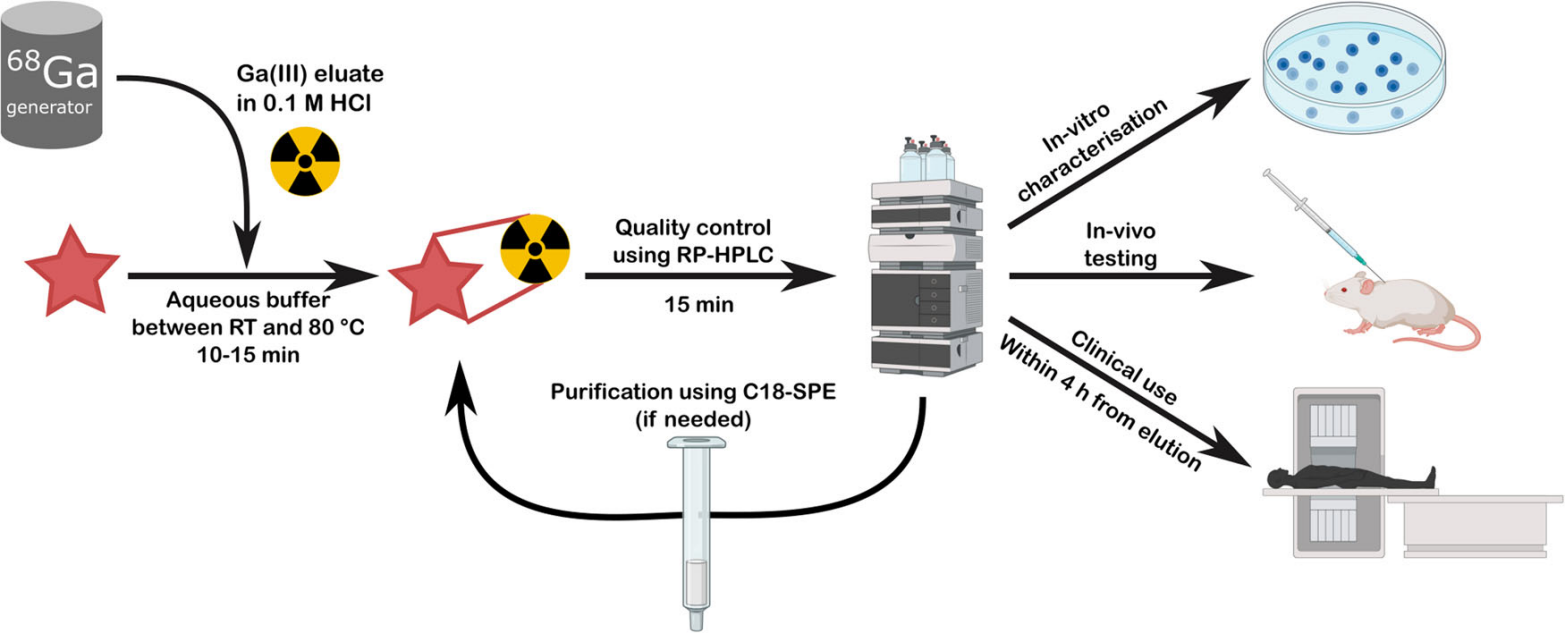
Timeline of chelator ^68^Ga radiolabelling. Gallium-68 (III) chloride eluate is mixed with the ligand under optimised conditions depending on the chelator chemistry. After analysis by reversed phase HPLC (RP-HPLC) or LCMS, the radio-pharmaceutical must be used within short timeframe due to the short half-life of ^68^Ga. (SPE solid phase extraction). Created with Biorender.

**Fig. 12:**
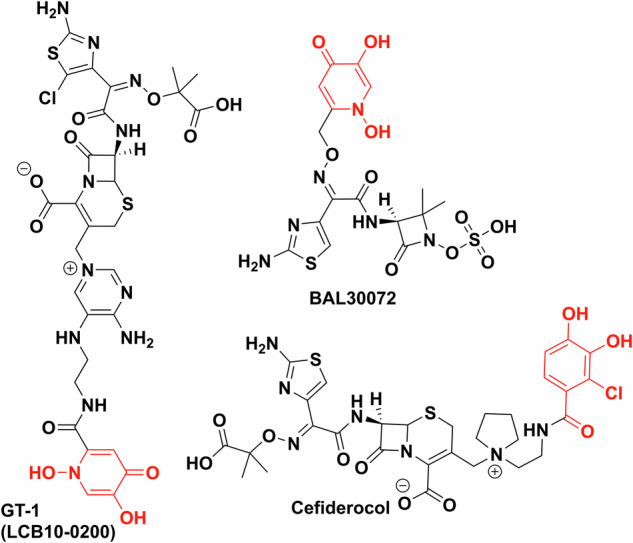
Trojan horse antibiotics towards the clinic. All are synthetic chelator–antibiotic conjugates. GT-1 and BAL30072 contain dihydroxypyridinone as the synthetic iron chelator (red), cefiderocol uses a chlorinated salicylic acid moiety (red). Chemdraw Professional version 22.2.0.3300.

Siderophore characterisation has often relied on their ability to chelate diamagnetic gallium (Ga^3+^) a property which facilitates NMR characterisation of a metal bound complex^129–131^. Gallium is particularly well suited for the replacement of the iron ion in siderophores due to its similar ionic radius, charge and relative lack of toxicity. In radioimaging, gallium-67 is being phased out due to its long half-life (78 h)^132^. However, gallium-68 has a half-life of just 68 min making it ideal for diagnostic imaging. The widespread adoption of the affordable and easy-to-use ^68^Ge/^68^Ga generators has provided a boost to the study of siderophores as gallium chelators and imaging agents (Fig. 11). Due to the short half-life of ^68^Ga, the synthesis of labelled chelators needs to be quick. Thus, chelation must be rapid, kinetically and thermodynamically favourable and high yielding enabling facile purification. For most of these procedures there is ~1 h between eluting radioactivity from the generator and using the hot imaging agent (Fig. 12). Three ^68^Ga compounds for diagnostics: ^68^Ga-DOTATATE, ^68^Ga-DOTATOC, ^68^Ga-PSMA-11, have been approved (FDA and EMA) in the last 10 years. While the ligands are not siderophores, their recent development indicates a growing field.

In 2010, a proof-of-concept study from Petrik et al. used siderophores triacetylfusarinine C (TAFC) and ferricrocin (FC) to chelate gallium-68 and image *Aspergillus* infections in lungs, including the important respiratory fungal pathogen *Aspergillus fumigatus*^133^. Both siderophores showed relatively good labelling characteristics, but TAFC, from *A. fumigatus*, proved to be slightly superior in vitro with virtually no unspecific uptake and slower exchange when incubated with excess cold Ga-siderophore complex. In uninfected BALB/c mice models, TAFC showed desirable characteristics such as rapid renal excretion and low levels in blood^133^. Ferricrocin C complexes were problematic with high blood and organ radioactivity levels, indicating the breakdown of the siderophore. Based on these results, TAFC was selected for imaging infected rat models with good results, indicating the siderophore-^68^Ga complex is selective, with rapid uptake in infected lung tissues and secondary uptake in the kidneys and the bladder^133^.

In follow-up studies, they demonstrated the selectivity of TAFC-^68^Ga uptake in different microorganisms and investigated the possibility of labelling other hydroxamate siderophores (coprogen, ferrichrome, ferrioxamine B, ferrioxamine E, fusarinine C) with ^68^Ga^134,135^.

Desferrioxamine (DFO), produced by many *Streptomyces* strains, has also been identified as a promising chelator to selectively image bacteria over mammalian cells^136^. In vivo studies with *P. aeruginosa* and *S. aureus* infected mice showed good uptake in infected tissue, with no off-target labelling except kidneys and bladder. Recently Bendova et al. used ornibactin, a siderophore from *Burkholderia* spp., to establish selective ^68^Ga based PET imaging of *Burkholderia cepacia* complex infected mice^137^. *B. cepacia* complex is a dangerous infection in immunocompromised patients, especially those with cystic fibrosis^138^. Preliminary data showed good radiolabelling purity (>95%) and stability in serum^137^. In vitro studies showed exceptional uptake in the parent bacterial strain and moderate-to-poor uptake in *S. aureus* and *P. aeruginosa*. Other tested strains (*E. coli, Streptococcus* spp*., Candida albicans, K. pneumoniae)* showed virtually no uptake. Biodistribution studies established that the compound is excreted through the kidneys and showed good targeting in infected mice model.

As mentioned above, ^67^Ga can also be used as a radiotracer. ^67^Ga citrate was shown to accumulate in tumours during clinical trials in the 1960s^139^. This work established the medical diagnostic technique called the gallium scan. While the use of gallium-67 scan has now largely been replaced by PET imaging, the isotope can still be useful as a longer half-life surrogate for gallium-68. The Boros group has done extensive work in radiolabelling DFO and linear desferrichrome using ^67^Ga^140^ showing that ciprofloxacin conjugates of these siderophores can be radiolabelled under routine gallium radiolabelling conditions and that the linear desferrichrome conjugate is a potent in vivo imaging and therapeutic agent^141^. Uniquely, they showed the curative effect of the desferrichrome–ciprofloxacin conjugate during in vivo mouse experiments^141^. The Boros, Duhme-Klair and Routledge groups explored a salmochelin-S4 fragment derivative in a similar ^67^Ga labelling experiment^94^.

### Transforming the clinic

Trojan horse antibiotics have been of significant interest to industry, but while several leads entered clinical trials and showed promising MICs, they have faced issues. These trials have primarily focused on conjugating a synthetic iron chelator with an already approved and a clinically used antibiotic. Dihydroxypyridinone and chlorinated catechol moieties are often the chosen synthetic iron chelators and siderophore mimics used to avoid metabolism by catechol *O*-methyltransferases and cytochrome P450^142,143^. BAL30072 (Basilea Pharmaceutica), a dihydroxypyridone conjugate of β-lactam, tigemonam, showed activity against about 70% of carbapenem-resistant *Enterobacteriaceae*, while also being a poor substrate for many β-lactamases, except for extended spectrum β-lactamase variants^144^. In clinical trials, however, it resulted in hepatoxicity due to inhibition of glycolysis (Fig. 12)^145^. LCB10-0200/GT-1 is also a dihydroxypyridinone–cephalosporin conjugate developed by LegoChem Bioscience and Geom Therapeutics^146^ which showed promising in vitro data against *P. aeruginosa* (MIC 0.5 mg/L vs 32 mg/L for ciprofloxacin), however, it also did not pass phase 1 clinical trials^147^. GSK3342830 (GSK and Shionogi) entered clinical trials in 2017 but unfortunately resulted in adverse effects and so was not continued (Fig. 12)^148^.

However, Shionogi’s cefiderocol, (previously S-649266) proved to be more efficacious in clinical trials than the best available therapy without significant adverse effects^149^. It was FDA approved in 2019 (EMA in 2020) making cefiderocol the first approved iron chelator–antibiotic conjugate and indeed a new class of approved antibiotics^150,151^.

The interest in these compounds as radio-diagnostic tools has been more subdued. This is due to the still relative high efficacy of existing antibiotics compared to antineoplastics, and generally the low requirement for high-resolution spatial information in diagnosing bacterial infections. However, with the rise in complex infections (e.g. XDR) the clinical need for rapid and relatively low-cost imaging diagnostics is likely to increase. Thus, there are two active clinical trials involving ^68^Ga-desferrioxamine adducts for PET imaging bacterial infections, under the EudraCT Number: 2020-002868-31 and NCT05285072.

## Conclusions

As AMR develops as a major health threat, bottlenecks in new antimicrobial development continues to exacerbate the problem. The recent addition of cefiderocol to the clinical arsenal of antibiotics is an important step and it validates the approach of utilising bacterial nutrient uptake systems as a method of selective drug delivery.

This work has been enabled by decades of research on understanding siderophore production and uptake in microorganisms inspiring the development of siderophore conjugates. However, to further develop this class of antibiotics, we require a greater fundamental understanding of the regulation of siderophore biosynthesis. In addition, elucidation of structures and mechanisms of the multicomponent uptake systems is needed employing, e.g. cryo-EM and computational modelling, respectively. Siderophore transporters are highly dynamic and thus we need a full picture of the molecular mechanisms of transport through the outer and inner membranes, in the case of Gram-negatives, to enable more rational design of conjugates and develop “design rules”.

In addition, we have a poor understanding of how siderophores are produced and utilised in mixed microbial populations such as the microbiome. Thus, population and coculture studies are required to understand how siderophores facilitate interactions between both infectious and commensal bacteria, as well as their role in host-pathogen interactions. This will likely require both wider study and improved modelling of nutrient and metabolite flux in microbial communities. It is probable that machine learning will play a role in understanding these complex chemical networks.

Greater understanding at the protein and cell levels will enable the identification of further therapeutic targets and the development of more selective antimicrobials. The identification of siderophores produced solely by pathogens could be important to enable sensitive diagnostic tools and pathogen identification in the presence of commensal bacteria.

The need for further therapeutic development is clear, thus the introduction of government initiatives for antibiotic development across the world is welcome. For example, in the UK the National Health Service’s new antibiotic subscription scheme funds antibiotic research and production. Similar measures are planned in the USA through the PASTEUR Act, and in the EU through the One Health AMR Candidate Partnership and should accelerate development in the field.

However significant challenges lie ahead. While the development and approval of cefiderocol is an important step forward, unfortunately, resistance to the drug was reported soon after entry to the market^152,153^. Resistance is proposed to occur via heteroresistance, whereby the majority of bacterial cells in the isolate are susceptible to the applied antimicrobial, but a small subpopulation of cells is resistant. This phenomenon often goes undetected during standard susceptibility testing^154^. This form of resistance, arising from the inherent heterogeneity of bacterial cultures, is unstable and poorly understood. Thus, there is a need to develop protocols to more readily detect it. Indeed, it illustrates the significant gaps in our understanding of bacterial virulence and resistance and the need for fundamental investigations into bacterial physiology alongside antimicrobial development.
